# Endovascular Treatment of Behcet Disease With Recurrent Infrainguinal Arterial Pseudoaneurysms: A Case Report and Literature Review

**DOI:** 10.1097/MD.0000000000003545

**Published:** 2016-05-13

**Authors:** Ze-yang Ding, Guan-nan Jin, Xi Ai, Li-yan Li, Ping Zheng, Yan Guan, Qi Wang, Zhi-wei Zhang, Jun Yang

**Affiliations:** From the Division of Vascular Surgery, Hepatic Surgery Center, Tongji Hospital (ZD, XA, LL, PZ, YG, QW, ZZ, JY); and Department of Nephrology, Union Hospital (GJ), Tongji Medical College, Huazhong University of Science and Technology, Wuhan, China.

## Abstract

Aneurysm or pseudoaneurysm formation is one of the vascular complications of Behcet disease. At present, the optimal treatment for the disease has not been established.

The authors report a case of vasculo-Behcet disease (v-BD) with recurrent pseudoaneurysms in the left infrainguinal arteries (common femoral artery, superficial femoral artery, and popliteal artery), as well as thrombosis in the popliteal vein and posterior tibial vein. The patient underwent 3 rounds of surgery, but developed a new pseudoaneurysm several months after each surgery. Eventually, the patient was successfully treated with a combination of endovascular repair, using a fully covered stent graft, and prednisone. The pseudoaneurysm regressed without recurrence for more than 1 year.

For v-BD, treatment with immunosuppressive therapy alone may not be sufficient to prevent the recurrence of pseudoaneurysms. For the endovascular treatment of pseudoaneurysms affecting the infrainguinal arteries in v-BD, a fully covered stent graft without oversizing is essential to prevent the recurrence of pseudoaneurysms.

## INTRODUCTION

Behcet disease (BD) is a type of systemic vasculitis characterized by oral and genital ulcers, uveitis, and a variety of skin and mucosal lesions. In addition, it can affect the vascular, gastrointestinal, and neurological systems.^1^ When the vascular system is involved in BD, it is termed as vasculo-BD (v-BD), which may present with thrombosis and superficial phlebitis in the venous system, and aneurysm, stenosis, and occlusion in the arterial system. The incidence of vascular involvement varies from 7% to 38% in patients with BD,^2^ and venous manifestations are more frequent than arterial ones.^3^ BD with arterial lesions showed poor prognosis in these patients due to the occurrence of complications such as rupture of aneurysm and arterial occlusion.^4^ To avoid these life-threatening complications, surgical intervention with graft interposition of these arterial lesions is necessary. However, postoperative complications, such as anastomotic pseudoaneurysm or occlusion of the stent graft, are frequently seen.^5,6^

In recent years, endovascular treatment of arterial lesions in BD has been performed, and the results showed that it was effective and safe, with an acceptable vascular complication rate and excellent patency of the treated site.^7^ Nonetheless, the outcomes of endovascular intervention in BD and the experience with stent graft placement for patients with BD still need to be investigated further.

In this paper, we report a patient with v-BD with recurrent pseudoaneurysms in the left infrainguinal arteries. This patient was eventually cured by endovascular treatment with an appropriate covered stent graft (CSG).

## METHODS

Ethical statement: this study was approved by the Ethics Committee of Tongji Hospital, Huazhong University of Science and Technology (HUST). As our case report does not violate the patient's privacy, informed consent is not necessary.

## CASE REPORT

**FIGURE 1:**
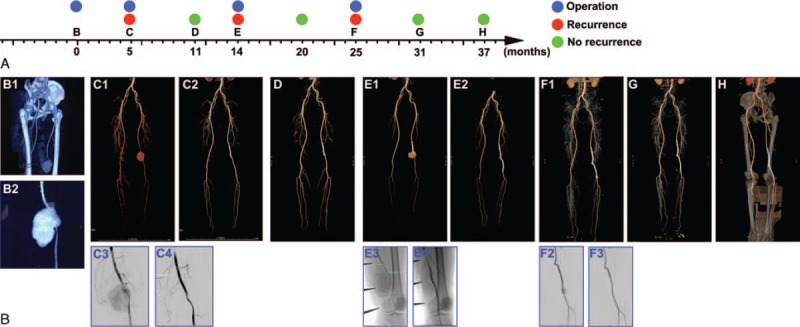
Endovascular treatment of BD with recurrent infrainguinal arterial pseudoaneurysms: a case report and literature review. (A) A sketched map with a time axis showing the therapeutic treatments and follow-up examinations of the reported patient with v-BD. Colored pie charts represent specific events (surgery, recurrence, or no recurrence) at each time point. (B–I) CT angiography of the infrainguinal arteries or arteriogram of the reported patient with v-BD at time points as indicated in (A). For Figure 1C, Figures 1C1 and 2 are the CT angiograms of this patient obtained before and after endovascular treatment, respectively. Figures 1C3 and 4 are the arteriograms obtained before (C3) and immediately after (C4) the implantation of the covered stent graft during the endovascular treatment of the aneurysm. Figures 1E and G, Figures 1E2, G2, E3, and G3 show the arteriograms obtained before (E2, G2) and immediately after (E3, G3) the implantation of the covered stent-graft during the endovascular treatment of the aneurysm. BD = Behcet disease, CT = computed tomographic, v-BD = vasculo-Behcet disease.

A 56-year-old Chinese man was admitted to our surgical ward because of a rapidly growing mass in the left thigh, with progressive and intermittent pain and numbness. The patient denied any history of trauma, drug abuse, or infective endocarditis. The patient was diagnosed as having BD a year before because of recurrent erythema nodosum, and oral and genital aphthous ulcers persisting for 1 year. In addition, at the time of diagnosis, the pathergy test result was positive, and laboratory test results showed erythrocyte sedimentation rate (ESR) of 84 mm/hour (reference range 0–15 mm/hour) and C-reactive protein (CRP) of 102.5 mg/L (reference range 0–8 mg/L). Antinuclear antibody and other autoantibodies, such as antineutrophil cytoplasmic antibody and anticardiolipin antibody, all tested negative. After diagnosis, immunosuppressive therapy using oral prednisone 20 mg and sulfasalazine 100 mg daily was initiated. A month later, the levels of ESR and CRP were gradually reduced to the normal range. The patient was then discharged on a regimen of prednisone 20 mg daily. A computed tomographic angiogram (CTA) and an ultrasonography revealed a left femoral artery pseudoaneurysm measuring 10 × 10 cm (Figure 1B). In addition, thromboses were found in the left popliteal vein and posterior tibial vein. Laboratory tests showed that the levels of ESR and serum CRP were within the normal range. The femoral artery pseudoaneurysm was soon resected, and graft interposition using an expanded polytetrafluoroethylene vascular graft was performed. Anticoagulant therapy (subcutaneous injection of enoxaparin, 4.25 kU per day for 15 days, followed by oral warfarin for another 6 months) was administered to treat extremity vein thromboses.

Five months later, the patient developed a symptomatic recurrence at the surgical site. A CTA revealed that there was a ruptured aneurysm of the left superficial femoral artery with active contrast extravasation (Figure 1C1). Thus, the patient was immediately admitted to our hybrid operating room, and an endovascular CSG (Bard Fluency, 8 mm × 8 cm) was deployed over the ruptured segment via the femoral approach. Completion angiogram (Figure 1C3) and CT angiography immediately after surgery (Figure 1C2) both revealed aneurysm exclusion without contrast extravasation. After this surgery, the patient was discharged on a regimen of prednisone 20 mg and aspirin 100 mg daily. Six months later, the patient remained asymptomatic. Follow-up CTA showed no residual aneurysm, endoleak, or any sign of migration or new aneurysm formation (Figure 1D).

**FIGURE 2:**
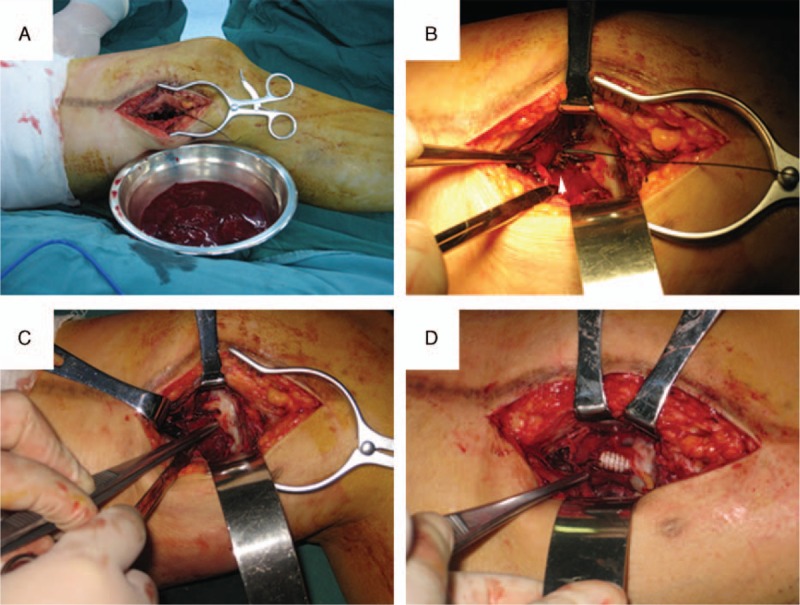
Intraoperative appearance during the 3rd surgery of the patient. (A) Clearance of the extravasated blood caused by rupture of pseudoaneurysm. (B) The structure of the artery wall at the ruptured site is completely disrupted, and the anchoring barb of the covered stent-graft is exposed (arrowhead). (C) Guidewire is inserted into the distal lumen of the artery. (D) After the repair of the pseudoaneurysm using a covered stent-graft.

However, 9 months after the 2nd surgery, the patient complained of a recurrent pulsating subcutaneous mass and pain in his left thigh. Furthermore, dyskinesia and dysesthesia occurred in his left crus. CT angiography revealed a ruptured aneurysm of the left superficial femoral artery, with a size of 7 × 5 cm (Figure 1E1). Moreover, an electromyogram revealed that neural injuries in his left tibial nerve and left peroneal nerve. Consequently, the patient underwent a 3rd surgery. During this surgery, endovascular treatment was initially attempted; however, the guidewire could not pass the ruptured aneurysm. Thus, we proceeded to expose the pseudoaneurysm, and surprizingly discovered that the structure of the arterial wall at the ruptured site was completely disrupted, and the anchoring barb of the CSG was exposed (Figure 2). We attributed the recurrence of the pseudoaneurym to the stimulation of the anchoring barb of the CSG to the arterial wall, and to the frequent motions of the knee joint. We opted to use a Gore Viabahn Endoprosthesis (VBC081502, 8 mm × 15 cm), instead of Fluency (8 mm × 8 cm) CSG, to repair the ruptured pseudoaneurysm. A completion angiogram (Figure 1E3 and 4) demonstrated aneurysm exclusion without contrast extravasation. After this surgery, the patient felt that the dyskinesia and dysesthesia of his left crus improved, and he underwent a rehabilitation program for 2 months at the Department of Rehabilitation of our hospital to address the neural injury. Six months after this surgery, both follow-up CT angiography (Figure 1F) and ultrasonic scan revealed that the pseudoaneurysm remained occluded, and there was no sign of leakage or endoleak.

Thirteen months after the 3rd surgery, the patient felt pain in his left thigh again, and repeat CTA revealed a pseudoaneurysm in the distal anastomotic site of his left popliteal artery (Figure 1G1). The patient was then subjected to another surgery. To reduce the irritation of the stent graft to the artery wall, we used a matched CSG without oversizing (Gore Viabahn VBC061502, 6 mm × 15 cm) to repair the ruptured segment via a femoral approach. A completion angiogram (Figure 1G2 and 3) demonstrated aneurysm exclusion without contrast extravasation. After this surgery, the follow-up CTA performed in both 6th (Figure 1H) and 12th month (Figure 1I) indicated successful patency without any sign of leakage or migration of the stent graft.

To date, the pseudoaneurysm has regressed without recurrence for more than 1 year and the patient continues to take prednisone 10 mg per day. The progression of v-BD in the case reported in this article is summarized in Table 1.

**TABLE 1:**
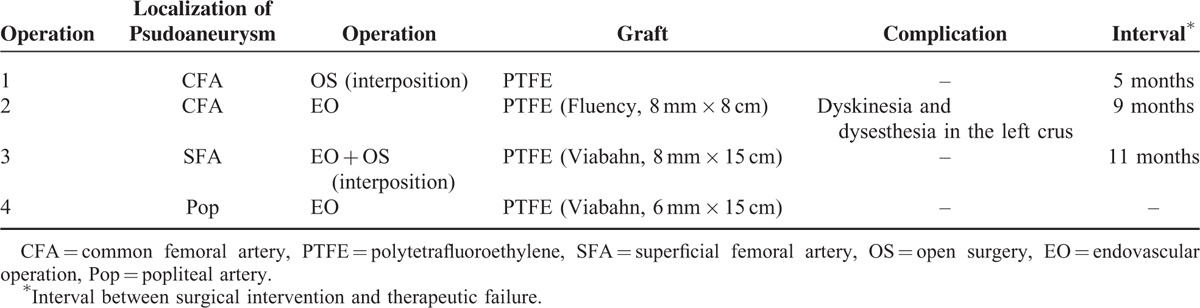
Progression of Disease in the Case Reported in This Article

## DISCUSSION

v-BD is a type of vasculitis involving both veins and arteries. This can lead to thromboses, occlusions, and aneurysms. The pathogenesis of v-BD is obliterative endarteritis involving the vasa vasorum of vessels. Thus, the arterial lesions of v-BD can affect all types of arteries. Previous investigations reported that in patients with v-BD, the aorta is the most common site of aneurysm formation, followed by the pulmonary and the femoral arteries.^8^ In addition, arterial aneurysms in v-BD are usually isolated and frequently coexist with venous thromboses.^9^ Here, we report a male patient who showed v-BD with pseudoaneurysms of the left femoral artery. In addition, thromboses in the popliteal vein and posterior tibial vein were also found in this patient. The patient was diagnosed as having BD 16 months prior to the onset of symptoms of an aneurysm in his left thigh. When diagnosed, the levels of CRP and ESR, which were tested to determine the activity of vasculitis, were higher than the normal range. Thus, this patient was treated with corticosteroids and immunosuppressive agents. The CRP and ESR levels returned to the normal range, suggesting that the activity of the vasculitis was controlled. However, 14 months later the patient developed another pseudoaneurysm of the left femoral artery, and the pseudoaneurysm recurred several times at the anastomotic site after surgery. During this period, the patient was under immunosuppressive treatment and the levels of CRP and ESR were within normal range. Previous investigations reported that based on the characteristics of vasculitis of v-BD, treatment of patients with v-BD with corticosteroids and immunosuppressive agents could effectively reduce the risk of development of anastomotic pseudoaneurysms after surgery.^10,11^ However, Kim et al^7^ reported that 2 among the 20 patients who received immunosuppressive therapy before endovascular treatment, with ESRs within the normal range, developed anastomotic pseudoaneurysms even after endovascular treatment. These reports are consistent with what our findings of this case. Although there is no specific laboratory marker for BD, previous studies used ESR and/or CRP as a marker for monitoring the active state of the disease.^12,13^ However, there is no consensus on the relationship between inflammatory parameters such as ESR or CRP and the severity or activity of vasculitis in v-BD. Taken together, these results suggest that in patients with v-BD, treatment with immunosuppressive agents is not sufficient to prevent the development of anastomotic pseudoaneurysms after surgery, and that the use of tests such as ESR and biomarkers such as CRP to determine the activity of vasculitis is of limited value.

Currently, there is no definitive therapeutic modality for v-BD with peripheral arterial lesions. We reviewed v-BD cases with infrainguinal arterial aneurysms or pseudoaneurysms that received surgical interventions in the English literature. The results are presented in Table 2. These results showed that open surgical intervention is the traditional treatment for v-BD with infrainguinal arterial aneurysms. However, anastomotic or new aneurysms, thromboses, and graft occlusions are the most frequently encountered complications after open surgical treatment of v-BD.^1^ Endovascular treatment of BD pseudoaneurysms is increasingly being performed in recent years, and results showed higher technical success rates and lower procedure-related complication rates when compared with traditional surgical interposition.^7^ Vasculitis is believed to be the pathogenesis of complications, such as aneurysm thrombosis and graft occlusion in patients with v-BD after surgery,^3^ and endovascular treatment of BD aneurysms can result in advantages of reducing the endothelial injury to the fragile arterial wall caused by vasculitis, and consequently decreasing the incidence of complications after surgery. In this case, the 1st surgery performed to treat that the femoral artery aneurysm was an open surgical interposition using a vascular graft; the aneurysm relapsed at the anastomotic site 6 months after this surgery. The subsequent treatments of the recurrent aneurysms were all via endovascular interventions, except the 3rd surgery, in which the endovascular guidewire could not be passed through the ruptured aneurysm successfully. The interval between endovascular treatments and therapeutic failures were 9 and 13 months. These results support the current view that endovascular intervention of BD aneurysms has a lower rate of anastomotic pseudoaneurysm formation than open surgery.

**TABLE 2:**
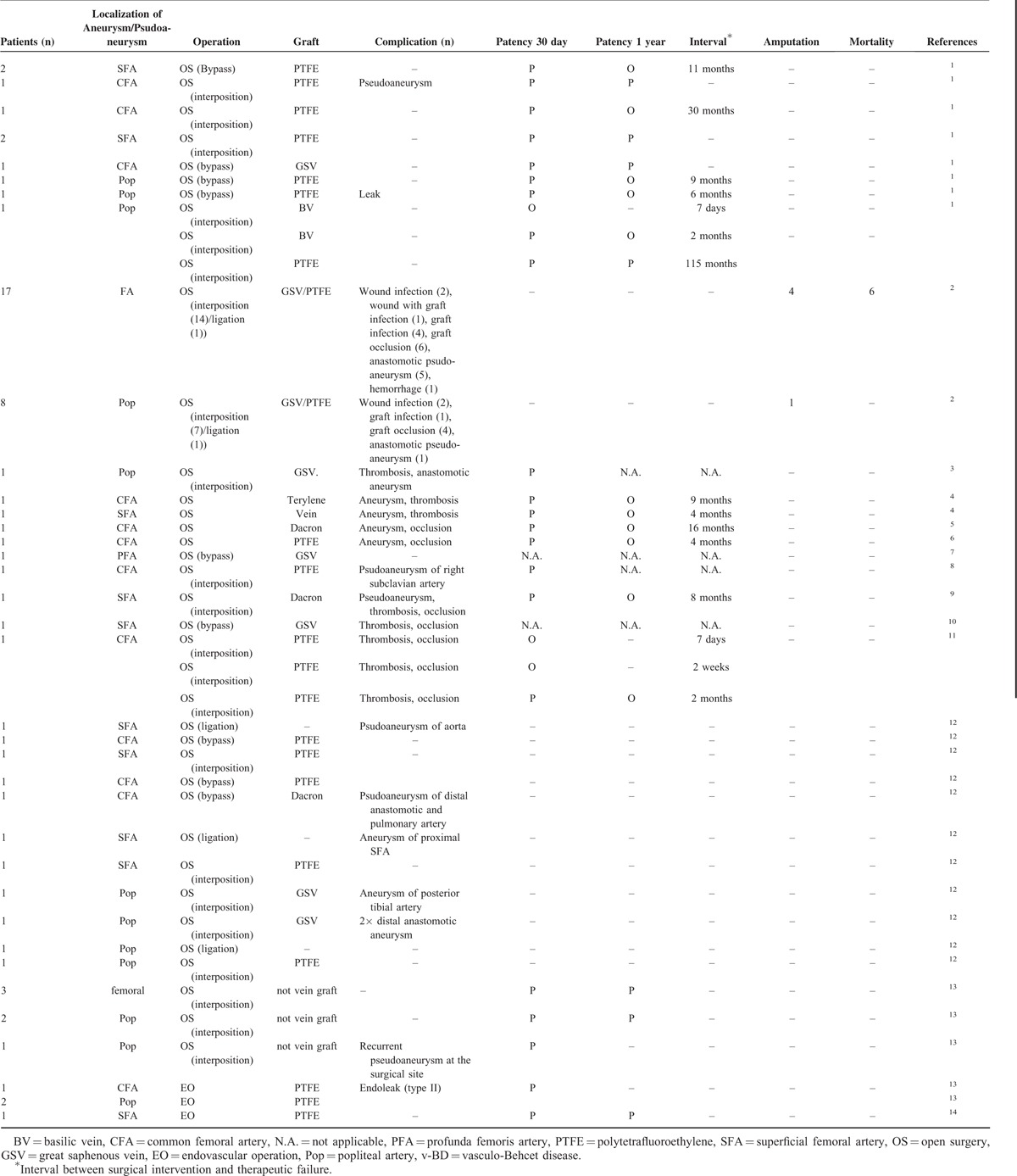
Reported v-BD Cases With Infrainguinal Arterial Aneurysms^7,12–24^

The recurrences of pseudoaneurysms over the anastomotic sites in this patient after stent-graft insertion gave us some suggestions in the selection of stent-grafts in the endovascular treatment of v-BD: in the first recurrence after endovascular intervention of the BD aneurysm, we found that the formation of an anastomotic pseudoaneurysm was possibly due to the chronic irritation of the fixing barb and naked edge of the Fluency CSG. Thus, we used the Viabahn CSG with its two edges covered to repair the aneurysm. Considering that the site of the ruptured pseudoaneurysm is near the knee joint, and frequent movement of the knee joint may aggravate the irritation in the arterial wall induced by the stent graft, we used the 15 cm Viabahn CSG, keeping the proximal and distal landing zones far from the inflamed ruptured site and knee joint. Although we carefully inserted this CSG to repair the ruptured BD aneurysm, the pseudoaneurysm recurred in the distal anastomotic site of the popliteal artery. To avoid endoleak and migration of the stent-graft, selecting a CSG with a diameter that is 10% to 20% larger than the diameter of the vessels (oversized) to the diameter of the vessels is the standard recommendation; however, the pressure of the oversized stent graft may damage the arterial wall of v-BD patients. To further reduce the irritation caused by the stent-graft to the inflammatory arterial wall, we used a matched Viabahn CSG without oversizing to repair the aneurysm this time. Although the risk of endoleak and migration of the stent-graft increased, the stent-graft has been patent for nearly 12 months, without any sign of migration or new aneurysm formation until present. Collectively, these results suggest that in the endovascular treatment of BD aneurysms, it is important to select an appropriate CSG to minimize the repeated endothelial injury caused by the stent-graft to the vasculitic arterial wall, and consequently reduce the recurrence of pseudoaneurysms.

## CONCLUSIONS

In conclusion, this case suggests that BD aneurysms might progress in patients with BD with normal CRP and ESR levels under immunosuppressive therapy. Endovascular stent-graft repair of BD aneurysms is a safe alternative to open surgical treatment. The irritation caused by a CSG to the vasculitic arterial wall is an important risk factor for the recurrence of anastomotic pseudoaneurysms in patients with BD after endovascular treatment. Consequently, fully CSGs with matched sizes and sufficient length to keep the fixing barb far from the inflammatory arterial wall of the aneurysm is recommended for endovascular repair of BD aneurysms.
